# COVID-19 outbreaks analysis in the Valencian Region of Spain in the prelude of the third wave

**DOI:** 10.3389/fpubh.2022.1010124

**Published:** 2022-11-17

**Authors:** David Fuente, David Hervás, Miguel Rebollo, J. Alberto Conejero, Nuria Oliver

**Affiliations:** ^1^Instituto Universitario de Aplicaciones de las Tecnologías de la Información y de las Comunicaciones Avanzadas, Universitat Politècnica de València, València, Spain; ^2^Departamento de Estadística e Investigación Operativa Aplicadas y Calidad, Universitat Politècnica de València, València, Spain; ^3^Valencia Research Institute on Artificial Intelligence, Universitat Politècnica de València, València, Spain; ^4^Instituto Universitario de Matemática Pura y Aplicada, Universitat Politècnica de València, València, Spain; ^5^ELLIS Alicante, Alicante, Spain

**Keywords:** COVID-19, SARS-CoV-2, epidemiological analysis, cluster, outbreak modeling, biomedical data science, Bayesian statistical model

## Abstract

**Introduction:**

The COVID-19 pandemic has led to unprecedented social and mobility restrictions on a global scale. Since its start in the spring of 2020, numerous scientific papers have been published on the characteristics of the virus, and the healthcare, economic and social consequences of the pandemic. However, in-depth analyses of the evolution of single coronavirus outbreaks have been rarely reported.

**Methods:**

In this paper, we analyze the main properties of all the tracked COVID-19 outbreaks in the Valencian Region between September and December of 2020. Our analysis includes the evaluation of the origin, dynamic evolution, duration, and spatial distribution of the outbreaks.

**Results:**

We find that the duration of the outbreaks follows a power-law distribution: most outbreaks are controlled within 2 weeks of their onset, and only a few last more than 2 months. We do not identify any significant differences in the outbreak properties with respect to the geographical location across the entire region. Finally, we also determine the cluster size distribution of each infection origin through a Bayesian statistical model.

**Discussion:**

We hope that our work will assist in optimizing and planning the resource assignment for future pandemic tracking efforts.

## 1. Introduction

Since March of 2020, the COVID-19 pandemic has put our society under tremendous pressure on a global scale, revealing vulnerabilities and pre-existing structural limitations in the public administrations and healthcare systems of most countries in the world. Unprecedented amounts of socio-sanitary and mobility data were made available to scientists, government officials, and decision-makers to inform and support their policy-making efforts (1). However, the quality of this information is generally low since it is often incomplete, noisy, has been originated by different methods and sources and is not systematically captured and shared for analysis (2–5). The global impact of the coronavirus pandemic has induced enormous research efforts by the scientific community, leading to hundreds of publications on this matter, from epidemiological (6–11), Bayesian (12), and machine learning-based (13, 14) computational models of the spread of the virus (13, 15–20), to reports of the pandemic's influence on the economy and psychology of the population worldwide (21).

Despite this wealth of COVID-19 publications, there are few reports about the characteristics and evolution of individual SARS-CoV-2 outbreaks within a wide region over a sustained time period. Most previous work on infection clusters has analyzed the evolution and characteristics of a single COVID-19 cluster within a social group (22–26). However, pandemic control efforts entail the early detection and modeling of the spread of the virus in all detected outbreaks, with the goal of isolating all infectious individuals and hence avoiding community transmission. Note that in the control phase of the pandemic, super-spreading events are of critical importance, since they might lead to community transmission.

In this paper, we focus on analyzing the origin, duration, spatial distribution, and temporal evolution of all tracked COVID-19 outbreaks in the Valencian Community of Spain for a period of 16 weeks between September 15th and December 29th, 2020. To the best of our knowledge, this is the longest study of SARS-CoV-2 outbreaks to date. The main research questions (RQ) that we address in our work are:

(1) **RQ1:** What is the dynamic evolution and duration of all the tracked COVID-19 outbreaks within the Valencian Region of Spain?;
(2) **RQ2:** What is the predominant origin of such outbreaks?;
(3) **RQ3:** Are there any differences in the outbreak characteristics among the 24 health departments in the region?;
(4) **RQ4:** What mathematical function best describes the relationship between the outbreak duration and frequency?
(5) **RQ5:** Can the cluster size distribution of each infection origin be modeled?

The paper is structured as follows: Next, we summarize the most related previous work. Section 2 describes the data used to model the COVID-19 outbreaks. The main results of our work are presented in Section 3, followed by our conclusions and lines of future research.

### 1.1. Related work

In this section, we describe the most relevant published works that study the evolution of individual COVID-19 outbreaks within a region or country.

Several contributions describe the number of cumulative cases of COVID-19 outbreaks resulting from the celebration of public events, such as religious gatherings (27), or outbreaks tracked in nursing homes (28). Other scientific works address multiple infection sources, such as in the workplace, leisure, educational and sanitary centers (29). For example, in Lakha et al. (30), most of the identified outbreaks started in workplaces, educational centers, and healthcare facilities, whereas the number of primary infections having a social origin was small.

Additionally, the clusters' size and their relationship with mortality rates in hospitals and other facilities in Japan have been analyzed in (31). In (32), the authors present the basic statistics of outbreaks in aged care facilities from North America, Europe, China, and Australia. An assessment of the cluster network of COVID-19 cases in Singapore up to March 2020 can be found in (33), where the authors report cluster sizes of fewer of four individuals in most of the cases. In (34), the authors outline the evolution of the emergence of COVID-19 infection clusters in Switzerland. They study the cluster duration and the viral load of the infected individuals. A systematic review of 65 articles conducted in (35) presents the outbreak size and origin of infection during the early stages of the pandemic in 2020 worldwide and highlights the importance of cluster transmission. In particular, most of the transmission chains had a familial origin, and their size was smaller than 10 cases, whereas the largest outbreak corresponded to a mass gathering in South Korea involving 112 people.

Some published works detail the size distribution of individual clusters at local, regional and even national levels. However, some of these contributions present aggregated data (36) that are only segmented by the infection origin but are, in reality, a collection of multiple clusters. Other research teams perform individual cluster analyses, but the number of reported clusters is rather small, being in the range of 10 to 200 clusters in any published study (37–40). Thus, these publications rarely describe large clusters, i.e., with more than four positive cases, and little information is provided on longer chains. However, large infection clusters and superspreading events have been argued to play a crucial role in the transmission of SARS-CoV-2 (29).

The overall duration of individual transmission chains in a small number of outbreaks is reported in a few publications (34, 39, 41) without presenting, however, the temporal evolution of the clusters. This lack of temporal outbreak data hinders a deeper understanding of the dynamics of the virus spread in the early stages of the outbreak before community transmission takes place. In addition, we only found comprehensive data describing how new cases appear within the same cluster for very specific groups, that is, for single clusters within a region. However, no work describes the temporal evolution of thousands of clusters, assessing their geographical and social context.

Mathematical tools can shed light on the intrinsic characteristics and dynamics of COVID-19 outbreaks. Several mechanistic models studying outbreak dynamics were already available before the COVID-19 pandemic, such as theoretical work based on stochastic Markov chain modeling for isolated populations (42). New models have been formulated and validated thanks to the availability of data during the current coronavirus pandemic. A mathematical model has been used to support the claim that outbreak clusters originating within schools in Canada could lead to average cluster sizes of more than 20 people if no social distancing measures were taken (37). The relationship between the distribution of outbreak sizes and their occurrence has been reported to follow a power-law distribution in (28, 43). In (44), there is evidence that cluster size of worldwide reported COVID-19 outbreaks follows a power law with respect to their rank size. The probability distribution of COVID-19 outbreak sizes in three Asian countries (Hong Kong, Japan, and Singapore) has been modeled as a negative binominal function (45). Similarly, a branching process model was applied to estimate outbreak size in multiple countries where the number of secondary transmissions was assumed to follow a negative-binomial distribution (46). In this regard, Nande et al. propose an interesting mathematical model of network transmission among social clusters (47). They found that the strength of within-household transmission is a fundamental determinant of the success in curbing the pandemic. Recent theoretical work has studied the transition from individual outbreaks to community transmission of the SARS-CoV-2 virus in Wuhan city during the first 2 months of 2020 (48). COVID-19 outbreak control is part of the widely adopted Test-Trace-Isolate (TTI) control strategy to avoid community transmission (49, 50). A comprehensive model on the effect of TTI on the virus transmission chains was recently published (51), and an empirical study of the effectiveness of TTI in Spain and Italy have been reported by De Nadai et al. (52).

Given all previously reported related work, the main contributions of this paper are three-fold. First, we study the main characteristics—namely duration, dynamics, origin source, and relation with other public health data—of 3,365 individual COVID-19 clusters tracked over 3.5 months in the Valencian Region of Spain. Second, we assess the mathematical properties of the cluster size distribution and third, we model the temporal evolution of the COVID-19 clusters *via* Bayesian statistics to better understand their dynamics and if there are disparities among the COVID-19 outbreaks with different infection sources.

## 2. Data and methods

### 2.1. Data description

**Figure 1:**
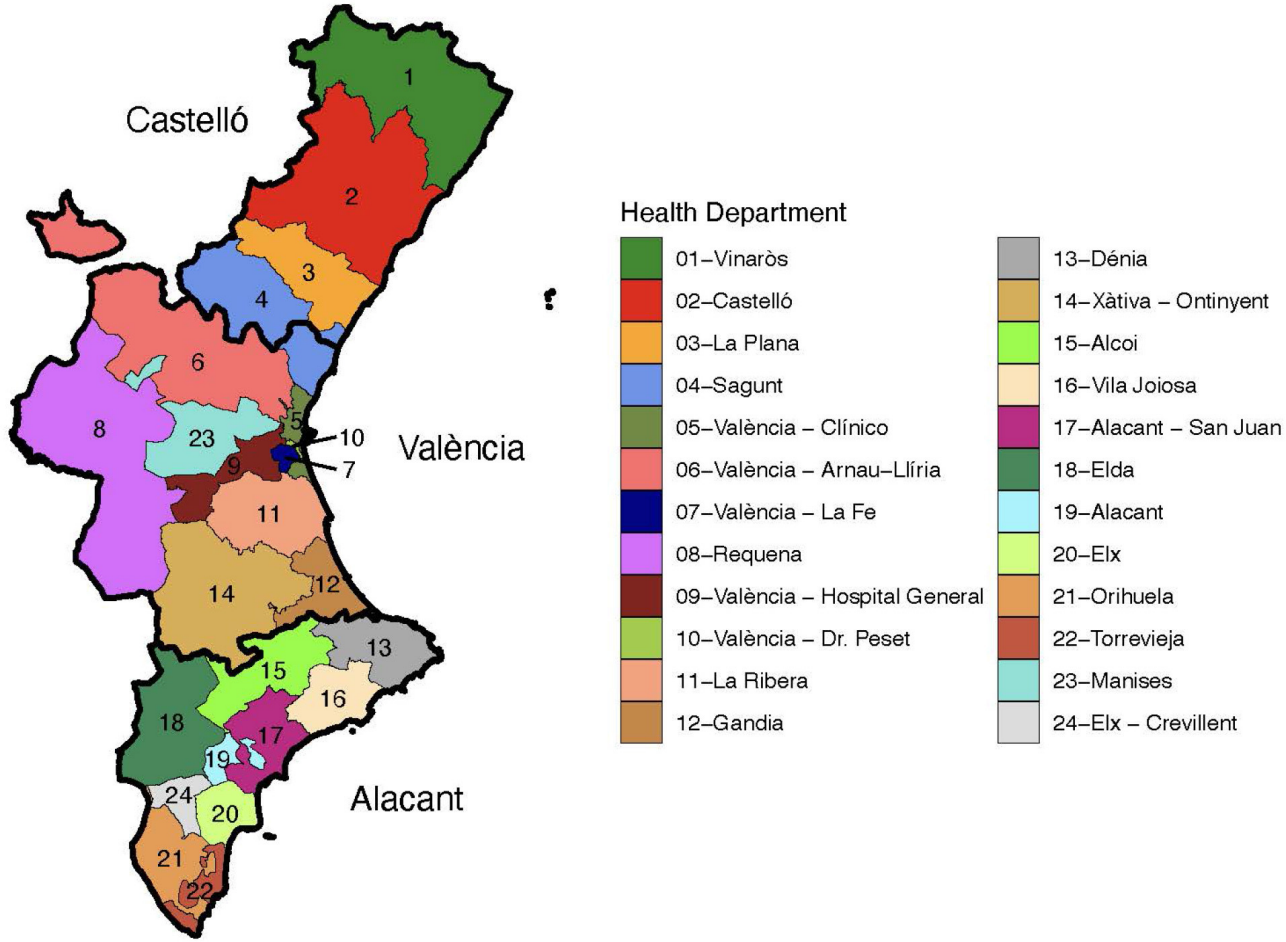
Health departments in the Valencian Region of Spain. Each province is depicted with a thicker contour and labeled.

The dataset analyzed in this paper consists of the temporal evolution of 3,365 COVID-19 outbreaks detected in the Valencian Autonomous Community or Region of Spain during the period of September 15th till December 29th, 2020. This region is the fourth most populous autonomous community in Spain after Andalusia, Catalonia, and Madrid, with more than five million inhabitants. Its capital, Valencia, is the third-largest city and metropolitan area in Spain. It is located along the Mediterranean coast on the East of Spain. . The Valencian Community consists of three administrative provinces: Castellón, Valencia, and Alicante. Their official name in Valencian language is Castelló, València, and Alacant. From a public health perspective, the Valencian Community is divided into 24 health departments (HD), which are the geographic areas served by a major hospital, as displayed in Figure 1. The distribution of HD by province reads as follows: Castellón (HD1 to HD4), València (HD4 to HD12, HD14, and HD23), and Alicante (HD13, HD15 to HD22, and HD24). It has to be noted that HD4 (Sagunt) comprises municipalities of two different provinces. Clusters of SARS-CoV-2 cases in our analysis involve a minimum of three cases, including confirmed close contacts with epidemiological linkage over a limited period of time.

The outbreak dataset comprises 16 weeks of outbreak information in the three provinces, 24 health departments and 230 municipalities of the region. It classifies the outbreaks in six different types depending on their origin: educational center, healthcare center, nursing home, vulnerable collectives (penitentiary centers and psychiatric hospitals), workplace, and social origin. The following variables are associated with each outbreak: outbreak identifier, outbreak origin, detection week, health department, municipality, province, and the number of diagnosed and suspected COVID-19 cases each week after the start of the outbreak.

This dataset was shared with the authors by the Public Policy and Analysis Directorate within the Presidency of the Valencian Regional Government, by virtue of a collaboration agreement between the Valencian Government and the authors in the context of the Data Science against COVID-19 taskforce which was established in March of 2020 and where the authors were members of. All the data is fully anonymized and in compliance with existing data protection regulations. The data sharing was approved by the Government's Data and Privacy Protection Officer. See also (53) for other studies of COVID-19 pandemic evolution in the Valencia region of Spain.

Next, we briefly enumerate the non-pharmaceutical interventions (NPIs) adopted in the Valencian Region during this period. We indicate the level of intensity of each applied NPI according to the COVID-19 Government Response Tracker (54): School closings were required at some educational levels (level 2 of 3); workplaces were closed for some sectors or working categories (level 2 of 3); public events were canceled (level 2 of 2); restrictions on gatherings of 10 people or less were implemented (level 4 of 4); a recommendation to stay at home was issued (level 1 of 3); restrictions on internal movements between regions/cities were deployed (level 1 of 2); and there was a ban on arrivals for international travelers from some regions in the world (level 3 of 4).

### 2.2. Data analysis methods

We first pre-processed the data to amend typographic mistakes and other sources of noise. For example, the number of new positive and suspected cases was wrongly annotated as the cumulative number was provided instead of the new positive and suspected cases. We transformed the three location variables (health department, municipality, province) and the outbreak origin into factors. We excluded the outbreaks labeled with the origin “other”, which corresponded to 22 outbreaks of the total number of 3,387.

Mean (standard deviation) and median (1st, 3rd quartile) values are reported in the case of numerical variables and relative and absolute frequencies in the case of categorical ones. We complement these basic figures with a variety of descriptive graphs, such as boxplots and scatterplots. The geospatial distribution of the outbreaks is depicted in choropleth maps of the Valencian Community. Statistical modeling of the evolution in number of cases of the outbreaks is performed using Bayesian negative binomial models with a monotonic effect for the week variable. Our models include each specific outbreak as a random factor with both a random intercept and a random slope for the week variable. The monotonic effect for the week variable is parameterized as introduced by (55), following Equation (1) which sets the linear predictor term of each observation as:


(1) $$\begin{array}{l} \eta_{n}=bD\overset{x_{n}}{\underset{i=1}{\sum }}\zeta_{i} \end{array}$$

where parameter ζ_*i*_ is a simplex (each value lies between zero and one and all sum to one), *D* is the number of unique values of the predictor minus one and *b* takes any real value and sets the global scale of the effect of the predictor on the response variable. *x* is the monotonic predictor (week) with *n* different observations. This method was proposed for modeling ordinal predictors in situations where their effects are assumed to be monotonic. Such models prevent an incorrect treatment of ordinal variables as nominal and avoid to overestimate the information provided by the variables. In the case of the COVID-19 pandemic, they have been used to estimate unreported COVID-19 deaths in the United States (56) and to measure the impact of COVID-19 vaccine misinformation on vaccination campaigns in the United Kingdom and the United States, too (57).

We provide 95% credible intervals for the estimate of each of the fitted models. Models were internally validated by computing the mean estimated Root-Mean-Square Error (RMSE) value using 10-fold cross-validation. All statistical analyses have been performed using R (version 4.0.1) and the brms (version 2.16.3) and clickR (version 0.8.0) R packages.

## 3. Results

In this section, we describe the main results of our analysis. We first present a general descriptive analysis of the data, followed by a temporal and spatial description of the outbreaks to address **RQ1** to **RQ3**. Next, we tackle **RQ4** and **RQ5** and model the characteristics of the outbreaks to shed light on their growth and the role that they play in the evolution of the pandemic.

### 3.1. Descriptive analysis

We analyze 3,365 tracked COVID-19 outbreaks in the Valencian Community of Spain. Table 1 depicts the basic statistics of the number of positive and suspected cases for each type of outbreak by origin of the infection. First, the distribution of the total number of cases per outbreak has a mean and a median value of 6 and 5 cases, respectively. Moreover, the maximum number of confirmed coronavirus cases in a single outbreak is 114 positive cases. Outbreaks originating in nursing homes had a notably larger size than any other type of outbreak: a mean of 13.9 and a median value of seven cases. Interestingly, this type of outbreak only represents 4.9% of the total number (165/3,365) but contributes with 11.0% of all the outbreak-related cases. This figure can be obtained by dividing the number of detected cases in the outbreaks of a given infection origin, i.e., nursing homes, by the total number of reported cases within all outbreaks. Education-related outbreaks have a median size of four cases, whereas the median of the other types of outbreaks is five individuals. Note that schools, high schools and universities were open in the region during the entire period of study, with in-classroom teaching in schools and high-schools, and hybrid (online plus in-classroom) teaching in universities. Suspected cases are also reported in Table 1, being the mean value very similar in outbreaks from all infection sources.

**Table 1:** Descriptive statistics of the outbreaks per origin of the infection.

	**Education center** **(*N* = 285)**	**Health center** **(*N* = 81)**	**Nursing home** **(*N* = 165)**	**Vulnerable c.** ***N* = 16)**	**Work-related** **(*N* = 557)**	**Social origin** **(*N* = 2,261)**	**Total** **(*N* = 3,365)**
**Positive cases**							
Mean (SD)	5.80 (3.99)	6.69 (5.42)	13.9 (17.4)	8.75 (8.52)	6.32 (6.80)	5.65 (3.33)	6.22 (5.95)
Median [Min, Max]	4 [3, 38]	5 [3, 34]	7 [3, 103]	5.5 [3, 33]	5 [3, 114]	5 [3, 49]	5 [3, 114]
**Suspected cases**							
Mean (SD)	15.8 (15.3)	17.3 (25.3)	13.0 (21.1)	18.1 (27.0)	15.0 (27.5)	13.8 (15.5)	14.2 (18.7)
Median [Min, Max]	12 [3, 133]	10 [3, 156]	7 [3, 209]	6.5 [3, 105]	10 [3, 560]	10 [3, 243]	10 [3, 560]

N, number of cases; SD, Standard deviation; Min, minimum; Max, maximum.

**Figure 2:**
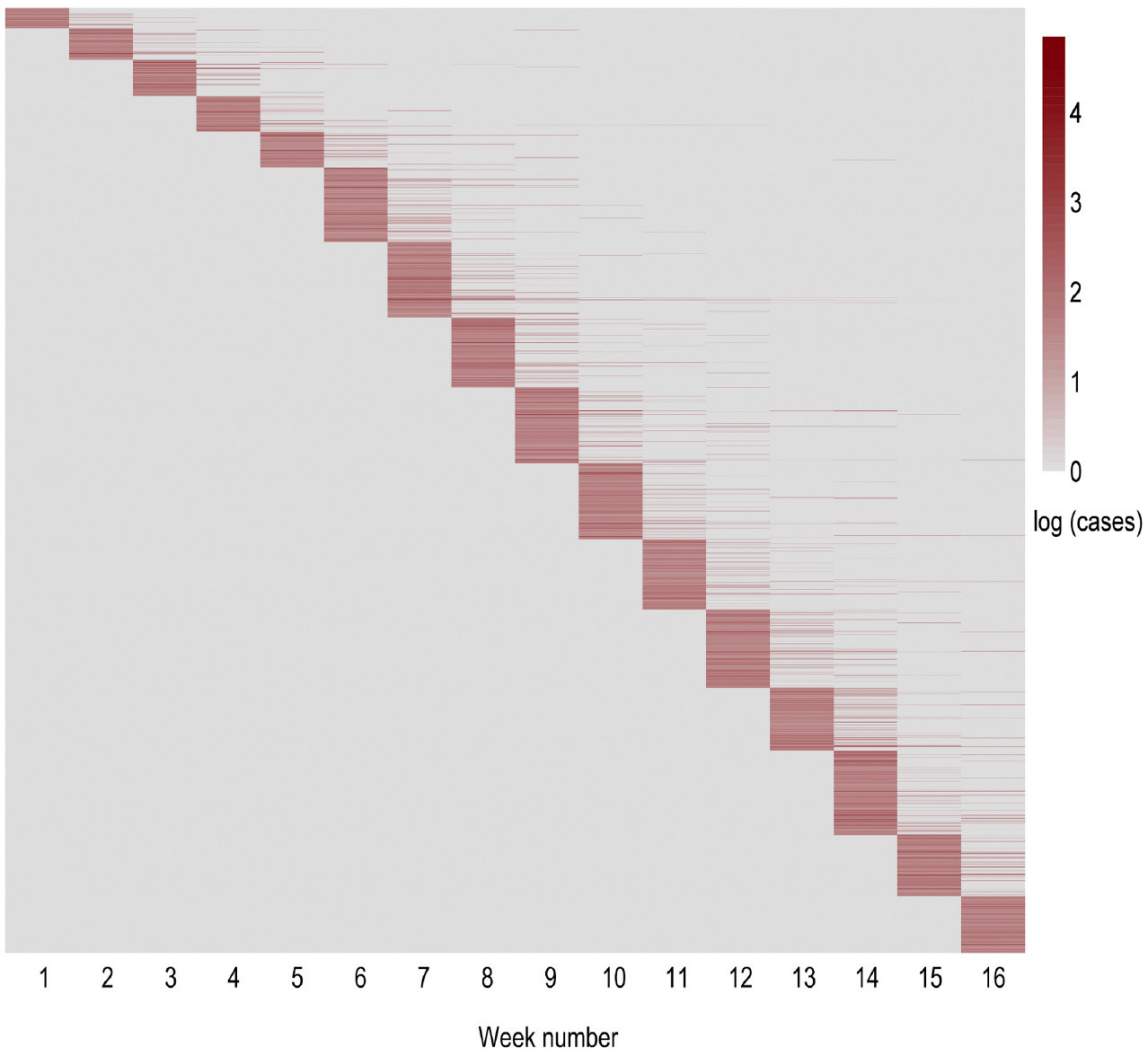
Heatmap of the weekly evolution of new positive cases within each outbreak in natural logarithmic scale. The horizontal axis indicates the evolution within the period of study in weeks. The vertical axis represents the order of appearance of each outbreak. Thus, each row displays an outbreak, while each column corresponds to the new cases reported in that outbreak in each of the following weeks (period of 16 weeks from September 15th till December 29th).

A heatmap visualization of the distribution of weekly new positive cases per outbreak is shown in Figure 2. Note how most outbreaks report cases during the first week in which they appear. We also observe an increase in outbreak detection on the sixth week of analysis, corresponding to the beginning of November. From that moment onwards, the number of newly identified outbreaks remains approximately constant. Most of the outbreaks report new cases in the initial 2 weeks after the first case has been identified, whereas less than 20 outbreaks display new cases over a period of at least 6 weeks.

**Figure 3:**
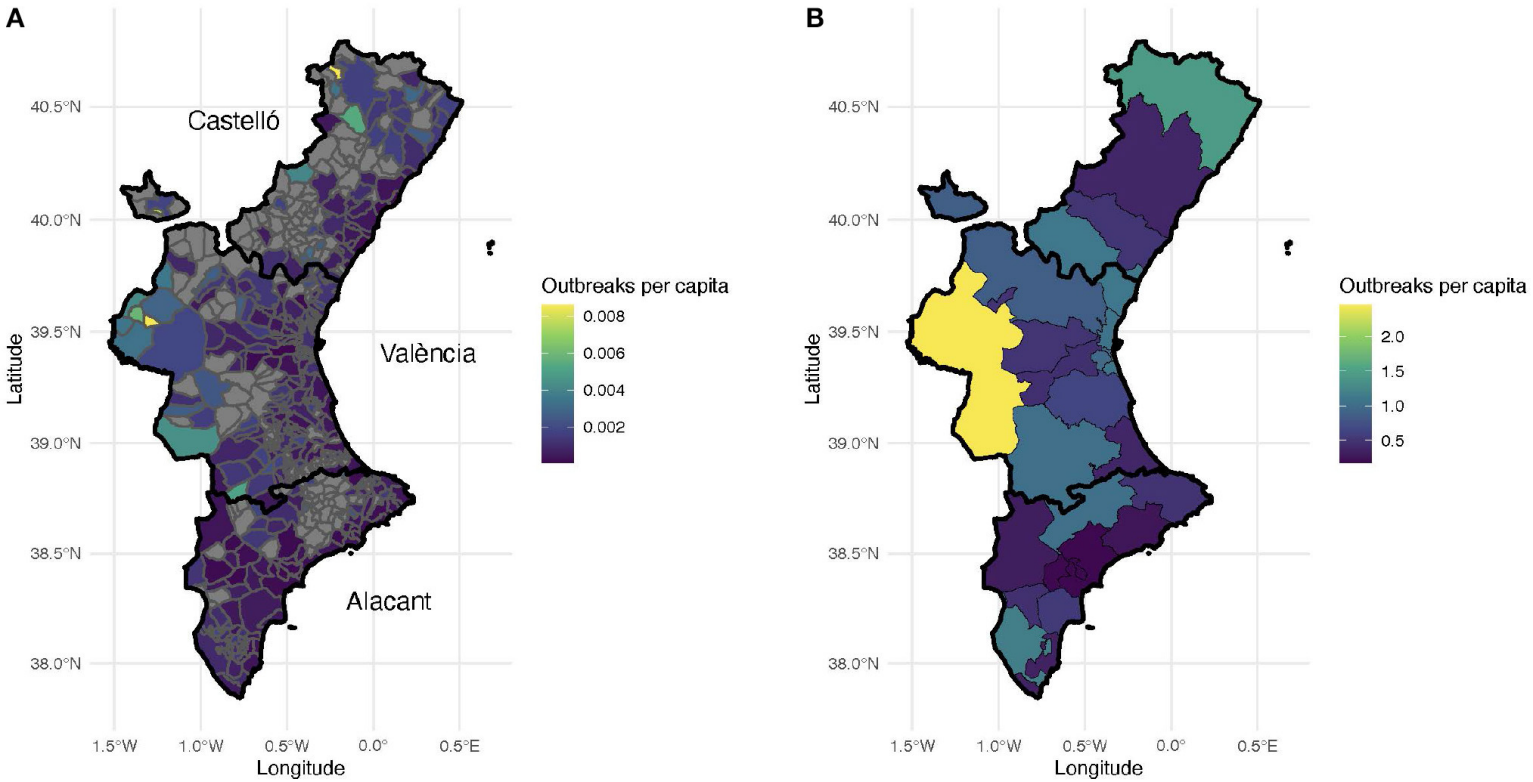
Number of outbreaks per thousand inhabitants. **(A)** Number of outbreaks per municipality. Municipalities without recorded outbreaks are displayed with gray color. Each province is outlined with a black contour. **(B)** Number of outbreaks per health department.

Geographically, almost two-thirds of the outbreaks (65%) were detected in the province of Valencia, 25% in the province of Alicante and roughly 10% in the province of Castellón. Note that the relative population size of these provinces is: Valencia (51%), Alicante (38%), and Castellón (11%). Thus, there was a larger presence of outbreaks in the province of Valencia than what one would have expected given its population. Figure 3 depicts a map of the Valencian Community with the number of outbreaks per capita in each municipality (Figure 3A) and in each health department (Figure 3B) during the period of study.

The areas without any confirmed coronavirus infections correspond to sparsely populated, rural municipalities in the interior of the Valencian Region. This is not surprising since most of these villages and small towns are located within forestry and hilly areas. The municipalities with the largest number of outbreaks per capita correspond to the three largest metropolitan areas in the region, namely Castellón (39°59'N 0°2'W), Valencia (39°28'N 0°22'W), and Alicante-Elche (38°20'N 0°29'W and 38°16'N 0°42'W, resp.). In general, more outbreaks are reported in the coastal areas than in the interior regions (Figure 3), probably due to larger population densities and tourism.

Remarkably, the number of outbreaks ranges between 0 and 2.5 clusters per thousand inhabitants. In addition, the median outbreak size in each HD ranged between 4 and 6 confirmed cases, except for the HD 19, Alicante, with a median value of 7 positive cases (data not shown). Moreover, there were no relevant differences among the health departments regarding the number and distribution of positive cases (data not shown).

**Figure 4:**
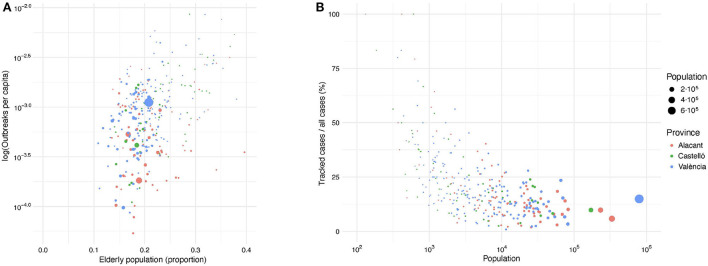
**(A)** Relationship between the number of outbreaks per capita (in logarithmic scale) and the percentage of population above 65 years old in each municipality. **(B)** Relationship between the ratio of confirmed cases within tracked outbreaks and the total number of confirmed COVID-19 cases in each municipality. Dot size and dot color correspond to the municipality population and province, respectively.

With respect to age, the SARS-CoV-2 virus is more likely to severely impact the elderly and individuals with compromised immune systems. Therefore, we analyzed the relationship between the percentage of elderly population (aged 65+ years old) in each province and the number of outbreaks per capita. As shown in Figure 4A, the distribution of elderly population in the region is quite homogeneous, independently of the municipality's size. Thus, this factor does not seem to have been a decisive variable to determine the number of outbreaks per capita. In Figure 4B, we observe that the larger the population of a municipality, the lower the percentage of tracked cases. Only in very small villages, more than 50% of the total cases were tracked. In large urban areas, healthcare resources and social conditions tend to be more homogeneous. Alternatively, small towns can be found in very different geographical environments when compared to large cities, i.e., coastal vs. rural regions. These geographic differences could impact the outbreak detection capabilities.

We also studied the relationship between the number of COVID-19 cases linked to outbreaks and all reported positive cases for each municipality in the Valencian Region. This relationship captures the mean coverage of confirmed cases of the outbreak tracking system with respect to all detected cases for each municipality (Figure 4B). There are no significant differences among the provinces, with larger cities having lower detection ratios than smaller municipalities. Intuitively, the larger the population in a municipality, the lower the coverage of the outbreak tracking system, converging to values close to 15% for the largest cities. This is probably due to a saturation of the contract tracing systems in such municipalities, as it has been previously reported (52).

### 3.2. Temporal analysis

**Figure 5:**
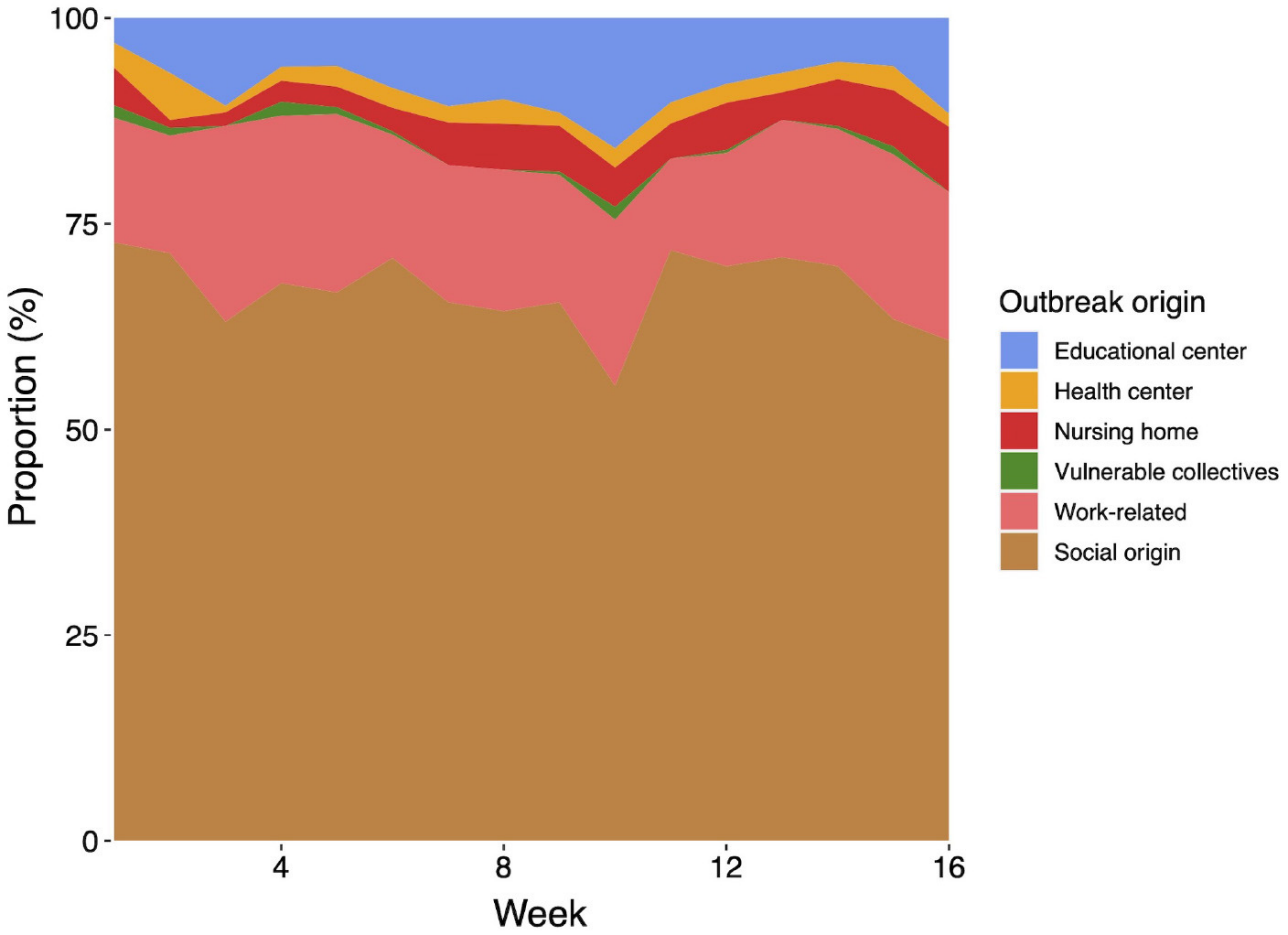
Distribution of the type of origin of the analyzed COVID-19 outbreaks throughout the period of study (16 weeks from September 15th till December 29th).

The temporal evolution of the type of origin of the COVID-19 outbreaks is shown in Figure 5. In our analysis, the proportion of newly confirmed cases for each infection source remains approximately constant during the assessed period. The most important source of infection is the social origin, with around 67% of all confirmed outbreaks. The distribution of the remaining outbreaks per type of origin is as follows: work-related (17%), educational center (8%), nursing home (5%), health center (2%), and vulnerable collectives (0.5%). Remarkably, few outbreaks were detected in healthcare facilities and nursing homes during the period of study, yet with a large number of infections, as previously described.

**Figure 6:**
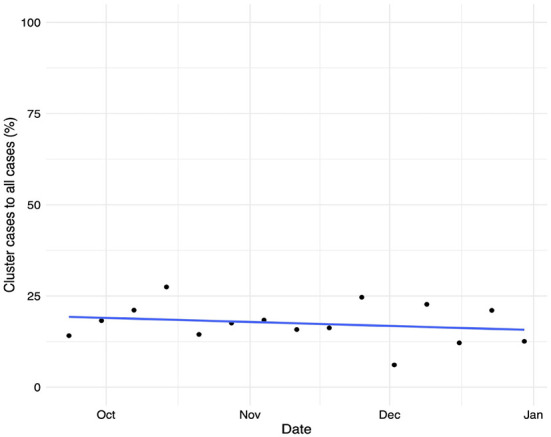
Temporal evolution of the proportion of confirmed cases linked to an outbreak vs. the total number of confirmed COVID-19 infections and its linear trend.

Figure 6 shows the temporal evolution of the total number of COVID-19 cases linked to an outbreak detected by the tracking system vs. the overall number of confirmed infections. We see that the number of cases linked to surveyed outbreaks accounts for less than 20% of the total number of cases, decreasing as the weeks progress. Note that the Valencian Community faced a second wave of COVID-19 infections in the Fall of 2020, followed by a severe third wave of infections after Christmas of 2020. The outbreak tracking system's coverage shows a slightly decreasing trend, with a coverage ratio fluctuating between 15 and 25% of the total number of detected cases. These figures are aligned with those reported in De Nadai et al. (52).

### 3.3. Outbreak modeling

In this section, we first study the relationship between the number of outbreaks and their duration. The latter is defined as the period of time (in weeks) between the outbreak identification and closure. This means between the week an outbreak is first identified and that when no additional cases have been reported for that outbreak. When this happens, the outbreak is labeled as closed.

**Figure 7:**
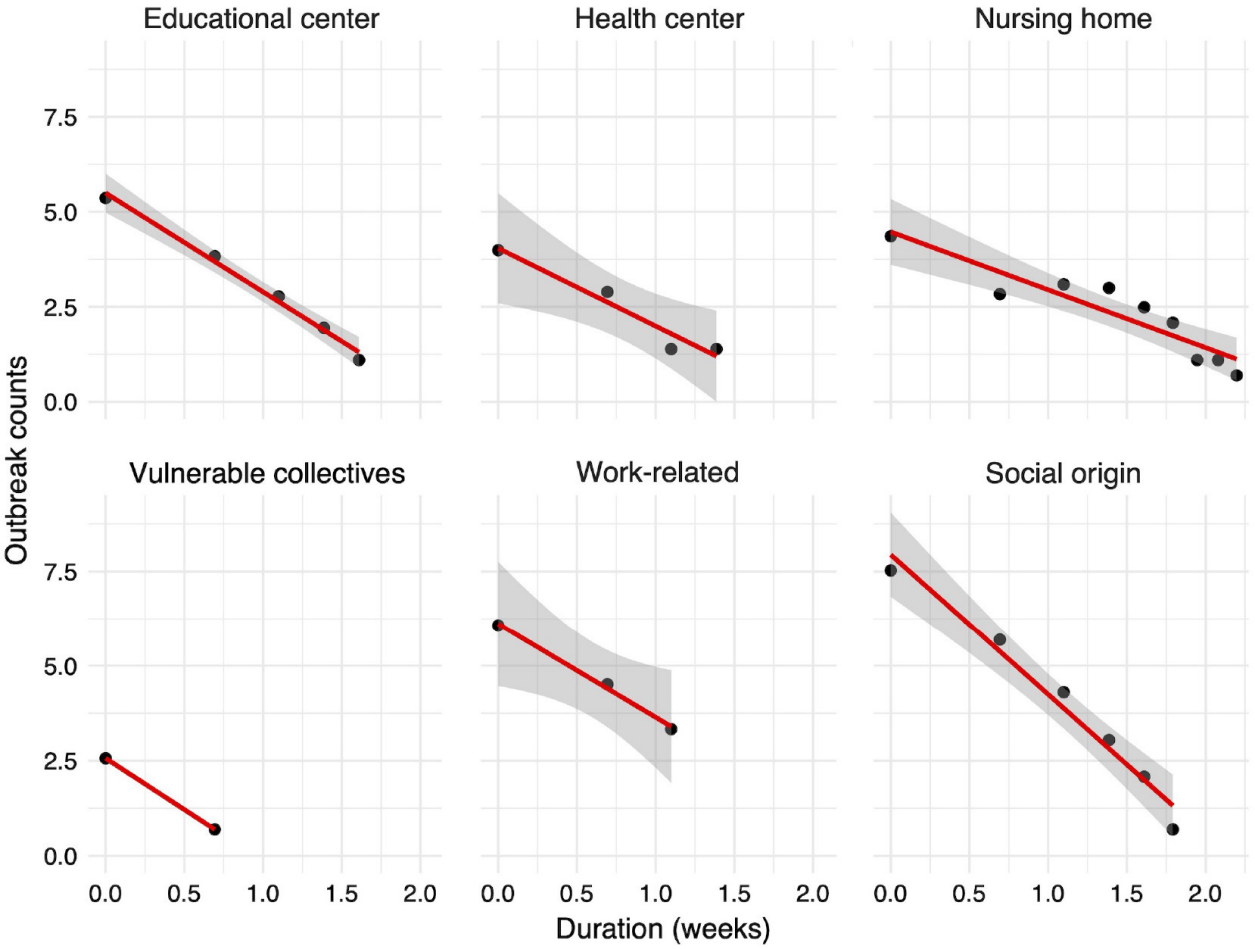
Relationship between the outbreak duration in weeks and the number of reported outbreaks (both in logarithmic scale). The 95% confidence interval around the linear regression line is shown with gray color. For the vulnerable collectives, no interval is shown as only two data points are available.

Interestingly, we find that this relationship follows a power law: most outbreaks last less than 2 weeks before they are controlled, whereas a few last for more than 2 months before they are fully contained (data not shown). Such pattern has been previously reported in (58–61). The estimate of the power law exponent is −3.4, and the adjusted R^2^-value for the linearized logarithmic values is close to 98%, indicating a strong power-law relationship between the duration and the number of outbreaks. The exponent is lower than −3, which is the lowest expected exponent in many natural and physical phenomena. This may be due to missing information and to the fact that parts of an outbreak may be reported independently. When we split the outbreaks by their infection origin, we also obtain power-law relationships as depicted in Figure 7. Outbreaks that occurred in healthcare centers and workplaces are controlled faster than outbreaks of social nature and those linked to nursing homes. According to our data, the most difficult outbreaks to control seem to be those detected in nursing homes.

**Figure 8:**
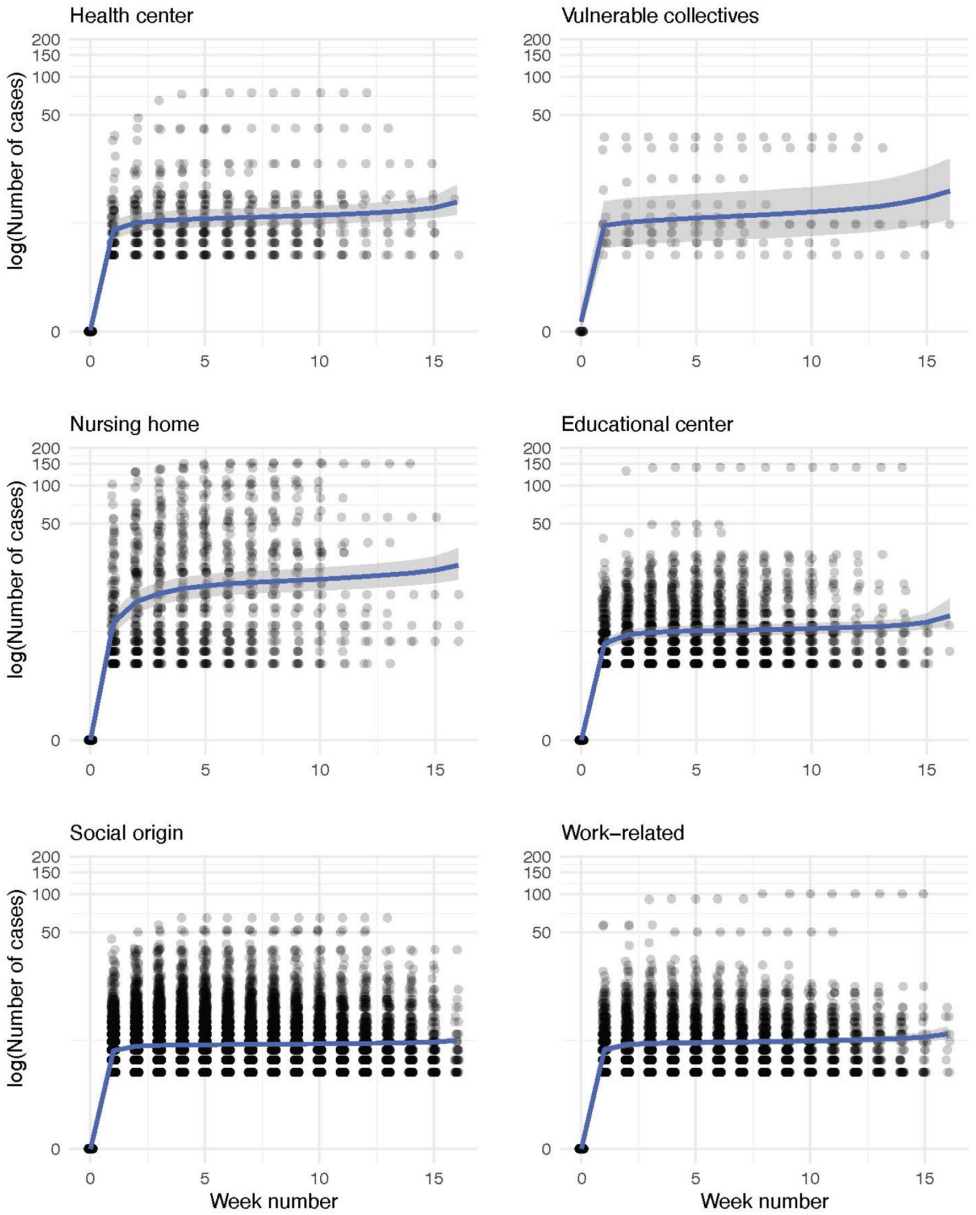
Conditional effect plots of the negative binomial models for the weekly evolution of the number of within-outbreak cases for each infection source. The number of cases of each outbreak is represented in a logarithmic scale (experimental data, shown as dots). Shadowed in gray, we depict the range that comprises the 95% credible interval of the expected value by the model, whose parameters are shown in Table 3.

Finally, we model the evolution of the outbreak cases for each infection source using a Bayesian negative binomial function with monotonic effects. The main statistics of each obtained distribution and the parameters of the model are shown in Tables 2, 3, respectively. The aim of this model is to predict the evolution of new cases within a detected outbreak and the corresponding credible interval. As shown in the Figure 8, all outbreak types display a common two-stage evolution pattern, with a sharp increase in the number of cases during the first 2 weeks followed by a stabilization with zero or negligible growth. The estimated total number of cases is the largest for outbreaks detected in nursing homes, as expected. Differences among other origin types are minor, being the health and educational centers types more prone to larger infection clusters. It is noteworthy that after the sharp increase of cases during the first weeks, the model predicts that the outbreaks will be controlled (hence, the small slope in the graph). We observe an apparent reactivation of each outbreak type, with the exception of those of social origin, in the last period (week 12 onwards). This apparent effect is due to the scarcity of data on outbreaks lasting more than 12 weeks.

**Table 2:** Main characteristics of the distribution of the expected number of cases for each type of COVID-19 outbreak.

**Outbreak type**	**Q1**	**Median**	**Q3**	**Mean**	**SD**	**Skewness**	**Kurtosis**
Health center	7	9	12	9.7	3.5	0.55	0.55
Nursing home	18	23	28	23.3	7.6	0.68	0.95
Vulnerable collectives	9	12	15	12.4	5.5	1.08	3.1
Educational center	6	8.5	11	8.8	3.3	0.54	0.52
Work-related	5	7	9	7.1	2.7	0.4	0.26
Social origin	4	6	8	6.2	2.5	0.38	0.09

Q1, first quartile of the data distribution; Q3, third quartile; SD, standard deviation.

**Table 3:** Main statistics of the monotonic effect model, i.e., a Bayesian negative binomial function with monotonic effects applied to each outbreak origin.

	**Estimate**	**Std. error**	**Exp (estimate)**	**Lower 95%**	**Upper 95%**
**Work-related**					
Intercept	−5.778	0.828	0.003	0.000	0.011
Week	0.483	0.052	1.621	1.496	1.828
**Educational center**					
Intercept	−5.167	0.808	0.006	0.001	0.020
Week	0.458	0.052	1.581	1.457	1.786
**Social origin**					
Intercept	−7.347	0.905	0.001	0.000	0.003
Week	0.573	0.057	1.773	1.628	2.020
**Health center**					
Intercept	−3.606	0.712	0.027	0.005	0.085
Week	0.366	0.045	1.443	1.341	1.599
**Vulnerable collectives**					
Intercept	−1.705	0.666	0.182	0.041	0.541
Week	0.261	0.043	1.298	1.209	1.427
**Nursing home**					
Intercept	−4.872	0.820	0.008	0.001	0.028
Week	0.500	0.051	1.649	1.520	1.856

The intercept estimate of infected people (at week 0), the week effect, and their standard deviation and the corresponding 95% credible interval are shown.

However, the modeled interval contains the horizontal trend without new cases in all outbreak sources. Thus, the outbreaks can be considered to be fully controlled in the last weeks. For social-origin and work-related outbreaks, the model seems to underestimate the number of cases. However, this is not the case as most of the outbreaks just contain less than 10 people. Conversely, we do not observe this effect for outbreaks with vulnerable-collectives and nursing home origins probably due to a larger variance of the number of cases in the case of outbreaks with these infection sources. Internal validation of the models using 10-fold cross-validation yielded the following RMSE values: 1.83 for the vulnerable collectives model, 1.19 for the social origin model, 2.78 for the health center model, 3.64 for the educational center model, 7.58 for the nursing home model, and 1.25 for the work-related model.

**Figure 9:**
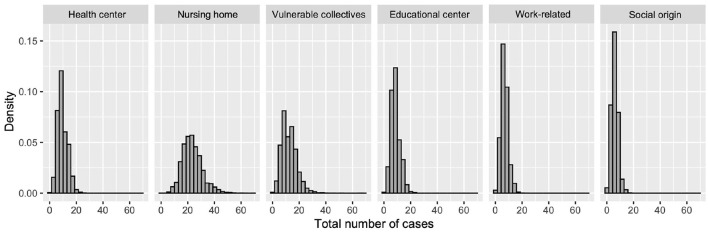
Probability histogram of expected cases for each type of outbreak origin.

We also estimate the probability distribution of the cluster size for each infection source using posterior draws from the posterior predictive distribution of each model. These distributions are displayed in Figure 9 and a detailed description of each of them is provided in Table 2. Outbreaks are expected to present less than 20 cases in five out of six infection sources, whereas the clusters reported in nursing homes are larger. According to the model, outbreaks in nursing homes could reach up to 40 cases.

## 4. Discussion and conclusions

In this paper, we have analyzed the characteristics of one of the largest COVID-19 outbreak datasets containing all the COVID-19 outbreaks reported in the Valencian Autonomous Community of Spain over a period of 16 weeks between September and December of 2020, right before the emergence of the third wave of COVID-19 infections in January-February 2021.

From our analyses, we draw several insights that could inform the design of public policies in future waves of this pandemic.

**1. Social and workplace infections are key:** Concerning the outbreak origin, most outbreaks are linked to social or workplace infections, with a contribution of 80% to the overall identified outbreaks. This is in line with the increased probability of infection in poorly ventilated indoor environments, especially inside buildings (62). Moreover, there is a small number of outbreaks in education centers, that is, not so many scholars were infected. This finding could be indicative of a successful deployment of the protocols implemented in education centers. Note that schools fully reopened in Spain in September of 2020 and were open during the entire period of study. These protocols entailed wearing facemasks in class, considering each primary school class as a social bubble, and reducing class sizes to respect at least 1.5 m distance between students. For those that were not able to attend in person due to COVID-19 quarantines, classes could be followed online.

Based on the social nature of most of the COVID-19 clusters in our dataset, it would seem advisable to strengthen communication campaigns and public policies aimed at informing the population about the transmission dangers of SARS-CoV-2 in social settings. Regarding workplace outbreaks, the region had well-defined workplace COVID-19 safety regulations. However, given our data, it seems that they might not have been rigorously complied with.

**2. Large metropolitan areas contribute to most outbreaks:** Geographically, the province of Valencia contributed to two-thirds of the total number of outbreak infections, as it is the largest, most densely populated metropolitan area in the region.

**3. All health departments behaved similarly:** We did not identify any significant differences in the structure and distribution of the outbreaks across the 24 health departments in the region. This homogeneity in the nature of outbreaks per health department is a consequence of the design of such health departments, covering similar types of populations across the region. However, the total number of outbreaks per capita was not homogeneously distributed at the municipality level, such that the metropolitan areas of the capital cities had a larger number of outbreaks per capita.

**4. Most outbreaks last less than 2 weeks:** More than 92% of the COVID-19 cases linked to outbreaks were controlled within the first 2 weeks. Remarkably, less than 1% of the outbreaks lasted for at least 2 months since the first case was detected. This means that the transmission chains seem to be properly contained given the adopted measures, e.g., the isolation of the confirmed cases. We found that the number of outbreaks follows a power law distribution with respect to their duration.

**5. The outbreak dynamics may be mathematically modeled:** We have modeled the outbreaks by means of a monotonic-effect Bayesian model. Our proposed approach could be relevant to support the work of contact tracers. The reproduction number and the efficacy of the contact tracing efforts will determine the parameters of the model.

Our predictions successfully capture the temporal dynamics of the six different types of outbreaks depending on their origin. According to our model, outbreaks linked to nursing homes and vulnerable collectives are expected to yield the largest number of confirmed infections and to last longer than outbreaks of other origins. Our modeling approach could be used to predict the expected number of cases and duration of new outbreaks, such as the right resources, e.g., contact tracers, hospital beds, and healthcare personnel that could be potentially allocated.

Moreover, we believe that the proposed model could be used to analyze outbreak data for other infectious diseases. However, the parameters of the model will depend on the specific virus, the target population, the applied non-pharmaceutical interventions and the efficacy of the tracing system. We hope that our analyses and outbreak models will help public health authorities to better track positive cases during future pandemics.

### 4.1. Limitations

Our work is not exempt from limitations. First, asymptomatic cases were not detected by the system and hence not included in our analysis. However, just a weekly update on the number of new cases is available. Hence, this weekly input might not provide enough temporal resolution to observe a smooth evolution of the growth of outbreaks that last less than 2 weeks. We have also detected noise in the reporting data: cases might be reported late and not annotated in the correct infection week. This leads to an artificial merging of cases from different weeks into a single data update.

The positive cases linked to outbreaks only account for 20% of the overall confirmed infections in the region, with a decrease in this ratio as the total number of COVID-19 cases increased toward the end of our period of study, when community transmission was a reality. A much higher ratio of tracked-outbreak cases to the total number of detected cases could have potentially delayed the start of community transmission.

Finally, our sample population, culture and behaviors might differ from those in other geographies and hence should be taken into consideration when applying our findings to other regions in the world.

## Data availability statement

The datasets presented in this article are not readily available because the data was accessible under an agreement signed with the Valencia Regional Government. Requests to access the datasets should be directed to General Directorate of Public Policy and Analysis of the Generalitat Valenciana.

## Author contributions

DF, MR, JC, and NO contributed to the conception and design of the study. JC organized the database. DF performed the statistical analysis and wrote the first draft of the manuscript. All authors contributed to manuscript revision, read, and approved the submitted version.

## Funding

NO has been partially supported by funding received by the ELLIS Unit Alicante Foundation from the Regional Government of Valencia in Spain (Generalitat Valenciana, Conselleria d'Innovació, Universitats, Ciència i Societat Digital, Dirección General para el Avance de la Sociedad Digital), by virtue of a collaboration agreement (Convenio Singular). MR, JC, and NO have been partially funded by grants from the BBVA Foundation through the IA4COVID19 research project and from the Valencian Government, grant VALENCIA IA4COVID (GVA-COVID19/2021/100) research projects, technological development, and innovation (R+D+i) by COVID-19.

## Conflict of interest

The authors declare that the research was conducted in the absence of any commercial or financial relationships that could be construed as a potential conflict of interest.

## Publisher's note

All claims expressed in this article are solely those of the authors and do not necessarily represent those of their affiliated organizations, or those of the publisher, the editors and the reviewers. Any product that may be evaluated in this article, or claim that may be made by its manufacturer, is not guaranteed or endorsed by the publisher.
